# Antimicrobial Activity and Protective Effect of Tuscan Bee Pollens on Oxidative and Endoplasmic Reticulum Stress in Different Cell-Based Models

**DOI:** 10.3390/foods10061422

**Published:** 2021-06-18

**Authors:** Morena Gabriele, Stefania Frassinetti, Laura Pucci

**Affiliations:** National Research Council, Institute of Agricultural Biology and Biotechnology (IBBA), Via Moruzzi 1, 56124 Pisa, Italy; frassinetti@ibba.cnr.it (S.F.); laura.pucci@ibba.cnr.it (L.P.)

**Keywords:** bee pollen, antioxidant activity, antihemolytic effect, Cellular Antioxidant Activity in Red Blood Cells CAA-RBC, antimicrobial activity, Minimum Inhibitory Concentration (MIC), Human Microvascular Endothelial Cells (HMEC-1), endoplasmic reticulum stress

## Abstract

Bee pollen is an apiary product of great interest owing to its high nutritional and therapeutic properties. This study aimed to assess the cellular antioxidant activity and the antihemolytic effect of *Castanea*, *Rubus*, and *Cistus* bee pollens on human erythrocytes. We also tested the antimicrobial potential of each sample on selected Gram-negative and Gram-positive bacteria. Finally, the effect of *Castanea* bee pollen, showing the best phytochemical profile, was analyzed on human microvascular endothelial cells exposed to thapsigargin, used as endoplasmic reticulum (ER) stressor. Our results showed good biological activities of all bee pollen samples that, under oxidative conditions, significantly improved the erythrocytes’ antioxidant activity and limited cell lyses. *Castanea* and *Cistus* showed comparable antihemolytic activities, with higher % hemolysis inhibition than *Rubus*. All samples exerted antimicrobial activity with different selectivity among all the tested microorganisms with minimal inhibitory concentration values ranging from 5 to 10 mg/mL. Finally, *Castanea* bee pollen was effective in reducing gene over-expression and oxidation process arising from thapsigargin treatment, with a maximum protective effect at 10 µg/mL. In conclusion, bee pollen represents a potential natural antibacterial and a good nutraceutical product useful in the prevention of free radical and ER stress-associated diseases.

## 1. Introduction

Apicultural products have been used for centuries in alternative medicine, in diets, or as dietary supplementation for their health and positive implications. Among others, bee pollen is an apicultural product that is receiving great attention as a functional food for its high nutritional value and therapeutic properties; indeed, it represents an important source of energy, bioactive compounds, and proteins for human nutrition [1].

Bee pollen results from the aggregation of collected flower pollens, nectar, and honeybees’ secretion in small colored pollen loads of one specific (monofloral) or more flower species (polyfloral). Currently, it represents the richest and most complete natural food supplying high levels of carbohydrates, proteins, enzymes, cofactors, unsaturated and saturated fatty acids, minerals, trace elements, essential amino acids, and vitamins, especially B, A, C and E [2,3,4].

Bee pollen is also an excellent source of bioactive compounds such as carotenoids and polyphenols, mainly hydroxycinnamic acid and flavonol glycosides and, likewise to the chemical composition, its phytochemical profile is affected by botanical origin, soil type, beekeeper activities, and climatic and preservation conditions [2,5,6].

The protective and therapeutic effects of bee pollen samples are related to the content and composition of polyphenol compounds that exert, among others, antioxidant, anti-inflammatory, antimicrobial, antifungal, anti-mutagenic, and antitumor effects [6,7,8,9]. Bee pollen is described as delaying aging, improves the immune, cardiovascular and digestive systems, and prevents prostate problems, arteriosclerosis, gastroenteritis, respiratory diseases, and osteoporosis by increasing bone mass [4,7]. Moreover, bee products are also reported to exert anti-angiogenic effects by preventing vascular endothelial growth factor (VEGF)-induced angiogenesis in human umbilical vein endothelial cells [10].

In a previous study, we investigated the botanical origin, the chemical and antioxidant compounds’ profile, and the free-radical scavenging activity of polyfloral Tuscan bee pollen composed of three botanical species, specifically *Castanea*, *Rubus,* and *Cistus* pollen. Based on these results, a strong in vitro antioxidant activity measured by Oxygen radical absorbance capacity (ORAC) assay and 2,2-diphenyl-1-picrylhydrazyl (DPPH) test was highlighted and, among samples, *Castanea* bee pollen showed the highest bioactive compounds content and a better phytochemicals profile [3].

To the best of our knowledge, to date no data on bee pollen effects on endoplasmic reticulum stress are available in the literature. The endoplasmic reticulum (ER) is a dynamic organelle involved in calcium homeostasis, and lipid and protein biosynthesis, as well as maturation and trafficking of several proteins [11]. ER stress is a condition occurring as a consequence of different stimuli, including viral infection, excess of lipids and glucose, accumulation of unfolded, misfolded, or mutated proteins, disturbances in cellular redox regulation, and reactive oxygen species (ROS) production. This condition elicits a protective/adaptive response known as unfolded protein response (UPR), which attempts to restore ER homeostasis; however, under severe and/or sustained signals, ER stress leads to activation of inflammatory, oxidative, autophagy, and cell death pathways [11,12,13,14]. An extensive cross-talk and interconnections between ER stress, inflammatory, and oxidative stress pathways occur in numerous pathological conditions, including atherosclerosis, diabetes, obesity, and cardiovascular diseases [11,12,13], and antioxidants can act in reducing both of them.

This study aimed to assess the antioxidant activity and the antihemolytic effects of *Castanea*, *Rubus,* and *Cistus* bee pollen on oxidized human erythrocytes by the CAA-RBC (cellular antioxidant activity in red blood cells) assay and the hemolysis test. We also tested the antimicrobial activity, expressed as the minimum inhibitory concentration (MIC), of each pollen sample on three Gram-negative (*Enterobacter aerogenes, Escherichia coli, and Salmonella enterica ser. Typhimurium*) and two Gram-positive (*Enterococcus faecalis* and *Staphylococcus aureus*) strains. Finally, we analyzed the effects of *Castanea* bee pollen, showing better phytochemicals profile, on the functional properties of human microvascular endothelial cells (HMEC-1) exposed to thapsigargin, used to induce ER stress.

## 2. Materials and Methods

### 2.1. Chemicals and Reagents

Quercetin dihydrate, 6-hydroxy-2,5,7,8-tetramethylchromane-2-carboxylic acid (Trolox), 2,7-dichlorofluorescein diacetate (DCFH-DA), 2,2-azobis (2-amidinopropane) dihydrochloride (AAPH), 3-(4,5-dimethylthiazol-2-yl)-2,5-diphenyltetrazolium bromide (MTT), thapsigargin, phosphate buffer saline (PBS), and dimethyl sulfoxide (DMSO) were purchased from Sigma-Aldrich (St. Louis, MO, U.S.). Ethanol and isopropanol were purchased from VWR (Radnor, PA, U.S.). Mueller Hinton Broth (MHB) and McFarland standard 0.5 were purchased from Oxoid (Basingstone, UK). All reagents, media, and medium supplements for cell culture were purchased from Sigma-Aldrich (St. Louis, MO, U.S.).

### 2.2. Plant Material and Extraction

Polyfloral bee pollen was collected during sunny days in Massa Macinaia (Lucca, Tuscany, Italy) between April and July 2013 using beehives equipped with bottom-fitted pollen traps. The fresh sample was stored at −20 °C in the dark until further analyses. Polyfloral bee pollen was a blend of 70% *Castanea* (yellow), 23% *Rubus* (green), and 7% *Cistus* (ochre) pollen [3] and was divided by color into three pollen samples. The extraction was carried out in triplicate, and bee pollen samples (50 mg/mL) were extracted with 95% ethanol while being shaken gently for 1 h at room temperature. Following 10 min centrifuge (Jouan CR3i centrifuge, Newport Pagnell, UK) at 2300× *g* at 4 °C, supernatants were collected, filtered (0.2 µm), and kept at 4 °C in the dark until use. The alcoholic extracts were lyophilized under vacuum, resuspended in DMSO 0.1% in water, and used on HMEC-1 cell culture.

### 2.3. Antibacterial Activity

#### 2.3.1. Pathogenic Bacterial Strains and Growth Conditions

Pathogenic bacterial strains from American Type Culture Collection (ATCC) were used: *Escherichia coli* (ATCC^®^ 25922™), *Salmonella enterica* *ser. Typhimurium* (ATCC^®^ 14028™), *Enterobacter aerogenes* (ATCC^®^ 13048™), *Staphylococcus aureus* (ATCC^®^ 25923™), and *Enterococcus faecalis* (ATCC^®^ 29212™). The above cultures were grown overnight at 37 °C under aerobic conditions on Mueller Hinton Broth (MHB).

#### 2.3.2. Minimum Inhibitory Concentration (MIC)

The minimum inhibitory concentration (MIC) of increasing concentration of each bee pollen samples (1, 2.5, 5, and 10 mg/mL) was determined according to Frassinetti et al. [15] on selected pathogenic bacteria, mainly three Gram-negative and two Gram-positive strains. Pathogenic microorganisms were maintained for 16 h at 37 °C in MHB; then, cultures were diluted to match the 0.5 McFarland standard turbidity. A typical mixture contained 50 µL of bacterial suspensions, corresponding to about 1–5 × 10^5^ CFU/mL MHB, 100 µL of bee pollen extract dilutions, and 100 µL of MHB in transparent sterile 96-well plates. Each plate was maintained at 37 °C for 24 h in aerobic conditions and a control, containing only the bacterial inoculum in MHB, was included on each one. Gentamicin and vancomycin were used as positive control (1 mg/mL in sterile physiological solution, corresponding to 0.05 mg/mL in the well). The optical density (O.D.) values were recorded at 600 nm, and the lowest concentration of bee pollen extracts able to suppress the microorganisms’ growth was defined as the MIC value.

### 2.4. Ex Vivo Biological Activities

#### 2.4.1. Preparation of Human Erythrocytes

Erythrocytes were collected from healthy blood donors upon informed consent for the use of residual blood for research purposes, according to the Italian regulations and, in particular, the regulations of “Fondazione G. Monasterio CNR-Regione Toscana”. Blood samples were collected in ethylene-diamine-tetra acetic acid (EDTA)-treated tubes and centrifuged 10 min at 2300× *g* at 4 °C. Following plasma and buffy coat removal, erythrocytes were washed twice with PBS pH 7.4.

#### 2.4.2. Cellular Antioxidant Activity (CAA) in Red Blood Cells

The antioxidant activity of ethanolic bee pollen extracts (100 μg/mL) was detected ex vivo on human erythrocytes under mild oxidation conditions as described by Frassinetti et al. [16]. Quercetin (8μM) was used as a standard, and the fluorescence was read at λ_ex =_ 485 nm and λ_em =_ 535 nm using a Victor^TM^ X3 Multilabel Plate Reader (Waltham, MA, USA). Each value was expressed according to the Wolfe and Liu [17] formula:

CAA unit = 100 − (∫SA/∫CA) × 100(1)

where ∫SA is the integrated area of the sample curve and ∫CA is the integrated area of the control curve.

#### 2.4.3. Erythrocytes Oxidative Hemolysis

The antihemolytic properties of increasing concentrations (20, 50, 100 and 200 μg/mL) of ethanolic bee pollen extracts were evaluated on oxidized human erythrocytes as described by Frassinetti et al. [16]. The erythrocytes hemolysis was induced by thermal decomposition of AAPH in peroxyl radicals and was spectrophotometrically recorded at 540 nm. Values were expressed as a percentage of hemolysis with respect to control corresponding to AAPH-treated erythrocytes.

### 2.5. Human Microvascular Endothelial Cells (HMEC-1) Treatments and Viability

The human microvascular endothelial cell (HMEC-1) line was obtained from the Centers for Disease Control and Prevention (Atlanta, GA, USA) and grown as previously reported [18]. A toxicity curve using increasing concentrations of *Castanea* bee pollen (1, 10, 100, and 200 µg/mL) and thapsigargin (0.01–3 μM) was performed. Further experiments were carried out on HMEC-1 cells stimulated for 2 h with or without 0.3 μM thapsigargin, following 1 h pretreatment with increasing concentrations of *Castanea* bee pollen (0–200 µg/mL). The cell viability at all treatment conditions was evaluated by the MTT assay as previously described [19]. The optical density was recorded at 540 nm through a multiplate reader (Multiskan EX, THERMO, Waltham, MA, U.S.) and reflected the amount of metabolically active cells.

### 2.6. RNA Extraction and Quantitative Real-Time PCR (qRT-PCR)

Total RNA was isolated using the RNeasy Mini Kit (Qiagen, Venlo, The Netherlands) and reverse-transcribed using the iScript^TM^ cDNA Synthesis Kit (Bio-Rad, Hercules, CA, USA). The quantitative Real-Time PCR was performed using the SsoFastTM EvaGreen^®^ Supermix (Bio-Rad, Hercules, CA, USA) in a CFX Connect Real-Time PCR Detection System (Bio-Rad, Hercules, CA, USA). Samples were assayed in triplicate, and the gene expression was calculated by the 2^−ΔΔCT^ relative quantification method. Gene primers were designed using Beacon Designer Sofware (PREMIER Biosof International, Palo Alto, CA, USA) as previously reported in Table 1 by Giusti et al. [20]. We used β-actin as the housekeeping gene.

### 2.7. Reactive Oxygen Species (ROS) Production

Cellular reactive oxygen species (ROS) were measured as previously described [21]. Cells were incubated for 30 min at room temperature in the dark with a DCFH-DA probe (15 µM/well), and intracellular ROS levels were detected using a VictorTM X3 Multilabel Plate Reader (Waltham, MA, U.S.) at λ_ex_ 485 nm and λ_em_ 535 nm.

### 2.8. Statistical Analysis

Results were expressed as mean ± standard deviation (SD) of at least three replicates. Differences between bee pollen samples were examined by one-way analysis of variance (ANOVA) with a Tukey’ or Dunnett’ multiple comparison test using GraphPad Prism version 5.00 for Windows (GraphPad Software, San Diego, CA, USA). A *p* < 0.05 was considered statistically significant.

## 3. Results and Discussion

### 3.1. Bee Pollen Antibacterial Potential

The Tuscan polyfloral bee pollen sample was composed of three botanical species corresponding to *Castanea* (70%), *Rubus* (23%), and *Cistus* (7%). In our previous work, we evaluated the chemical and phytochemical profile as well as the *in vitro* antioxidant activity of each botanical species, and we performed, for the first time, the front-face fluorescence spectroscopy as a fast tool to analyze the profile of bioactive compounds of each pollen type [3]. Based on our previous results *Castanea* bee pollen contained significantly higher levels of polyphenols (24.75 ± 0.78 vs. 21.19 ± 0.24 and 13.53 ± 0.4 mg GAE/g fw), flavonoids (15.86 ± 0.62 vs. 14.21 ± 0.56 and 5.91 ± 0.27 mg CE/g fw), and anthocyanins (77.37 ± 2.55 vs. 57.19 ± 5.84 and 53.44 ± 2.36 mg C3GE/L) than *Cistus* and *Rubus* species, respectively [3].

In the present study, the antibacterial potential of *Castanea, Cistus,* and *Rubus* bee pollen extracts at increasing concentrations (1, 2.5, 5, and 10 mg/mL) was tested on selected pathogenic bacterial strains and the relative bacterial growth values were showed in Table S1. As a comparison, the bacterial growth was monitored in the presence of the gentamicin and vancomycin antibiotics. 

The MIC value was used as a parameter of bacterial growth inhibition of bee pollen extracts, and results are listed in Table 1. All bee pollen extracts exerted antimicrobial activity with different selectivity among the tested microorganisms and MIC values ranging from 5 to 10 mg/mL; as expected, the standards gentamicin and vancomycin showed lower MIC values (0.05 mg/mL) than all bee pollen extracts.

Our results showed MIC values lower than those found by Cabrera and Montenegro [22], who analyzed the antimicrobial activity of Chilean bee pollen on human infectious microorganisms by qualitative (agar diffusion) and quantitative methods (minimum inhibitory and bactericide concentration). In this work, Cabrera and Montenegro [22] observed a different susceptibility of tested infectious agents, with the Gram-positive bacteria *S. aureus* and *Streptococcus pyogenes* more sensitive to the Chilean bee pollen extract and the Gram-negative bacteria *E. coli* and *P. aeruginosa* more resistant to it, with MIC values of 82.4 mg/mL for *E. coli*, 41.2 mg/mL for *P. aeruginosa*, and 20.6 mg/mL for *S. aureus* and *S. pyogenes*.

Our findings revealed that the most sensitive Gram-negative bacteria were *E. coli* ATCC 25922 and *S. typhimurium* ATCC 14028, showing a MIC value of 10 mg/mL for the *Castanea* and *Cistus* bee pollen samples, whereas *S. aureus* ATCC 25923 was inhibited at 5 and 10 mg/mL by the *Cistus* and *Castanea* extracts, respectively. While *Cistus* bee pollen exhibited antibacterial action against all tested bacteria, *Castanea* inhibited selectively *E. coli, S. typhimurium*, and *S. aureus* growth. In contrast, *Rubus* bee pollen was effective only on the Gram-positive strains (*S. aureus* ATCC 25923 and *E. faecalis* ATCC 29212) herein tested, with MIC values of 10 mg/mL of *Rubus* extract. Further, the Gram-negative strain *E. areogenes* ATCC 13048 was selectively inhibited only by the *Cistus* bee pollen with a MIC value of 5 mg/mL. A similar result was also observed by Morais et al. [9] screening the antimicrobial properties of five Portuguese bee pollen samples that, depending on the microorganism and the pollen type, selectively impaired the growth of yeasts and Gram-positive and Gram-negative bacteria. Further, using the disk diffusion assay, Carpes et al. [23] observed a different susceptibility to the Brazilian pollen types and ethanol concentration of extracts by tested microorganisms, mainly *Staphylococcus aureus* ATCC 25923*, Bacillus cereus*, *Bacillus subtilis* ATCC 21332, *Pseudomonas aeruginosa* ATCC 15442 and *Klebsiella sp.* In addition, the *Cistus* bee pollen analyzed here inhibited the growth of all Gram-negative and Gram-positive bacterial strains, with the last ones more sensitive than the former. Indeed, Gram-negative bacteria appeared more resistant to the *Cistus* bee pollen with MIC values superior than those obtained for the Gram-positive ones (10 mg/mL vs. 5 mg/mL, respectively), and this is probably due to the presence of cell wall and a generally more complex chemical structure [9].

*Castanea* and *Cistus* bee pollen exhibited a larger antimicrobial potential than the *Rubus* one, and this could be linked to a greater amount of polyphenols, mainly hydroxycinnamic acids and flavonoids [3]. These results are in line with those obtained by Pereira et al. [24] and Estevinho et al. [25] on walnut (*Juglans regia* L.) leaves and Northeast Portugal honey samples showing a positive relation between antimicrobial activity and total phenolic compounds.

Nevertheless, in some cases, the antibacterial activity did not correlate to the total phenolic compounds but was dependent on the nature of bioactive components present in extracts. Among others, Carpes et al. [23] detected a very low antimicrobial activity in Parana pollen extract at 60% of ethanol despite the fact that it contained the highest polyphenols and a degree of antioxidant activity above 80%. A similar result was also observed by Morais et al. [9], who detected no phenolics concentration dependence and found the greater antibacterial potential against microorganisms in the Portuguese bee pollen extract from the Parque Natural do Montesinho containing the lowest total phenols content.

### 3.2. Bee Pollen Samples Protect Human Erythrocytes from Free Radicals Damage

The antioxidant properties of *Castanea*, *Rubus*, and *Cistus* ethanolic extracts were screened on human erythrocytes under oxidative conditions using the CAA-RBC and the hemolysis tests. Erythrocytes have a key role in the body protecting from antioxidant and anti-inflammatory insult, and having neither nucleus nor mitochondria, depict as a valuable ex vivo cellular system to assess the radicals’ scavenging activities of several natural compounds [26]. In addition, the erythrocyte membrane contains both polyunsaturated fatty acids and proteins highly susceptible to oxidation [27].

Both the CAA-RBC and hemolysis tests are based on the use of oxidizing agent AAPH that by thermal decomposition in peroxyl radicals causes damage to the erythrocytes’ membrane through lipids and proteins peroxidation and, at high doses, the erythrocyte cells lysis.

**Figure 1:**
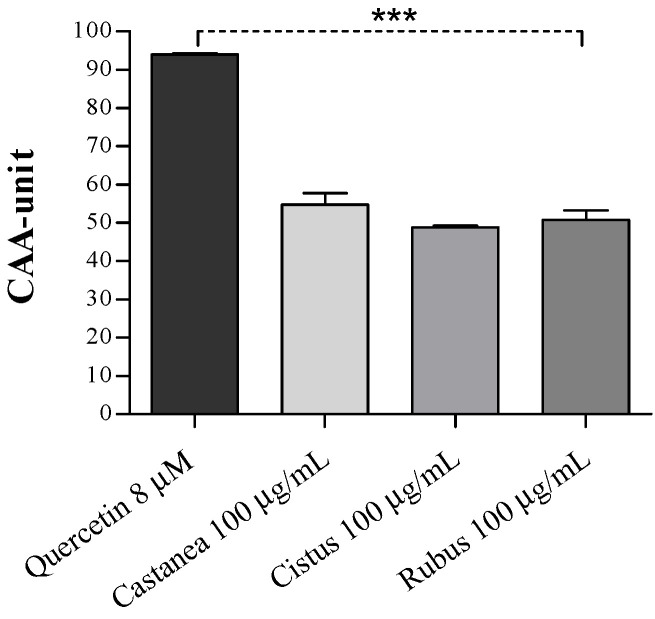
Effect of *Castanea*, *Cistus,* and *Rubus* bee pollen extracts (100 μg/mL) on the cellular antioxidant activity (CAA) of oxidized human erythrocytes. Quercetin (8 μM) was used as the reference standard. Results were expressed as mean ± SD. One-way analysis of variance (ANOVA) with Tukey’s multiple comparison test: * significantly different from control cells (AAPH-treated cells, CAA = 0), *** *p* < 0.001.

Herein, following 1-h pretreatment with each bee pollen extract (100 μg/mL), human erythrocytes were exposed to a low AAPH concentration (1.2 mM) to induce mild oxidation. As shown in Figure 1, all bee pollen pretreatments significantly improved the antioxidant activity of human erythrocytes by about 50% compared to the control (AAPH-treated cells, CAA = 0; *** *p* < 0.001), with CAA values lower than the quercetin (~92%) used as a standard. Moreover, no significant differences in the CAA values among all the analyzed pollen types were found.

**Figure 2:**
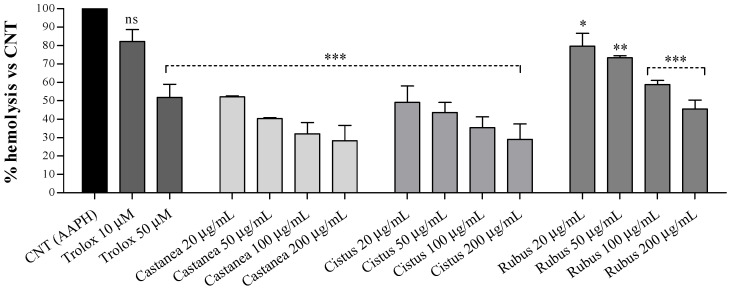
Effects of increasing concentrations (20, 50, 100, and 200 μg/mL) of *Castanea*, *Cistus* and *Rubus* bee pollen extracts on erythrocytes AAPH-induced oxidative hemolysis. Trolox (10 and 50 μM) was used as a standard. Results were expressed as mean ± SD. One-way ANOVA with Tukey’s multiple comparison test: * significantly different from CNT (AAPH-treated cells), * *p* < 0.05, ** *p* < 0.01, *** *p* < 0.001. ns: not statistically significant.

Further, the antihemolytic effect of all bee pollen species was tested on human erythrocytes exposed to a higher AAPH concentration (50 mM) responsible for the erythrocytes lysis. As shown in Figure 2, all bee pollen pretreatments exerted a dose-dependent hemolysis inhibition compared to erythrocytes exposed to the oxidizing agent alone (AAPH-treated cells).

Our results demonstrated comparable antihemolytic activities following the *Castanea* and *Cistus* pretreatment, with higher percentages of hemolysis inhibition than *Rubus* bee pollen at similar doses. Moreover, *Castanea* and *Cistus* extracts from 50 to 200 μg/mL showed greater antihemolytic effects than both doses of trolox (10 and 50 µM) used as a standard. Following human erythrocytes incubation with 200 μg/mL of *Castanea* and *Cistus* extracts, we observed about 71 ± 8.3% hemolysis reduction versus 54.4 ± 4.7% of *Rubus* extract at the same dose. Similar hemolysis inhibition profiles were observed at all tested concentrations, and these results are probably related to the greater polyphenols, flavonoids, and flavonols levels detected in the *Castanea* and *Cistus* bee pollen than in the *Rubus* ones [3].

Very few studies focusing on the biological and antioxidant protection of human erythrocytes by bee pollen and apiary products are available in the literature. Among them, we recently tested the antioxidant protection of 10 bee pollen samples of different botanical origin (e.g., *Erica*, *Eucalyptus*, *Prunus*, *Brassicaceae*, *Asteraceae* T., *Rubus*, *Rosa*, *Trifolium pretense*, and *Viburnum*) from Lucca and Massa Carrara provinces (Tuscany, Italy) on human erythrocytes under mildly oxidative condition [28]; for the first time, this study revealed good cellular antioxidant activity following 1 h pretreatment with 50 µg/mL of bee pollen extracts with CAA values ranging from 27.22 ± 6.99 to 54.61 ± 8.51 for *Erica* and *Eucalyptus*, respectively, and results are in line with those herein discussed.

Moreover, our results are in accordance with those reported by Araújo et al. [29] on 9 bee pollen extracts of different botanical origins (e.g., *Cocos nucifera*, *Miconia*, *Spondias*, *Eucalyptus*) showing high protection against AAPH-induced erythrocytes lysis, with a 44–86% hemolysis reduction depending on the bee pollen species. Similarly, Campos et al. [30] showed a dose- and time-dependent antihemolytic activity of Brazilian *T. febrigi* propolis ethanolic extract with a 46 ± 3.6% of hemolysis reduction at the highest tested concentration (125 μg/mL). A similar result was also observed by Valente et al. [27] on Portuguese propolis samples that strongly protect erythrocytes from hemolysis in a time- and dose-dependent manner with IC_50_ values of 6.3 ± 0.7 and 10.4 ± 2.7 μg/mL for propolis samples harvested in the Bornes and Fundão regions, respectively. As previously reported by Valente et al. [27], the protection of the erythrocyte membrane from the AAPH-induced hemolysis is ascribed to the propolis polyphenols fraction able to counteract the peroxyl radicals generated by the oxidizing agent and to inhibit the oxidation of lipids in the erythrocyte membrane. Likewise, our results showed better hemolysis protection following erythrocytes incubation with *Castanea* and *Cistus* bee pollens, richer in polyphenols.

Therefore, our findings showed a significantly higher cellular antioxidant activity following all bee pollen pretreatments and a better erythrocytes hemolysis protection by *Castanea* and *Cistus* bee pollens, suggesting good ex vivo biological activity as free radical scavengers and natural antioxidants.

### 3.3. Castanea Bee Pollen Counteracts Thapsigargin Induced-ER Stress in HMEC-1 Cells

The endoplasmic reticulum homeostasis is finely regulated, and a chronic excess of several signals such as ROS overproduction, viral infection, and abnormal accumulation of mutated/misfolded/unfolded proteins, which trigger a protective/adaptive response known as “unfolded protein response (UPR)”, can elicit ER stress if they persist over time. Recent evidence suggests that the ER stress response, besides apoptosis, activates inflammatory, oxidative, and autophagy pathways [12]. Inflammation and oxidative stress are important triggers of vascular endothelial dysfunction and activation [31]. Moreover, as described by Gotoh et al. [12], C/EBP-homologous protein (CHOP), the ER stress-responsive transcription factor, is also involved in several inflammatory and metabolic processes and plays a relevant role in the onset and progression of many metabolic and cardiovascular diseases.

An extensive cross-talk and interconnections between ER stress, inflammatory, and oxidative stress pathways occur in numerous pathological conditions [11,12,13], and diets enriched in antioxidants can be effective in reducing them.

To the best of our knowledge, for the first time, we aimed to investigate the protective effect of *Castanea* bee pollen, which exhibits the highest phytochemical content among bee pollen samples herein analyzed, in human microvascular endothelial cells (HMEC-1) under ER stress conditions by evaluating cell viability, intracellular ROS production, and the expression of factors involved in ER stress, endothelial activation, and inflammation. To identify the optimal treatment condition and detect possible cytotoxic effects, we first performed a toxicity curve using increasing concentrations of *Castanea* bee pollen (1–200 μg/mL) and thapsigargin (0.01–3 μM), a plant-derived sesquiterpene lactone, used to induce ER stress.

**Figure 3:**
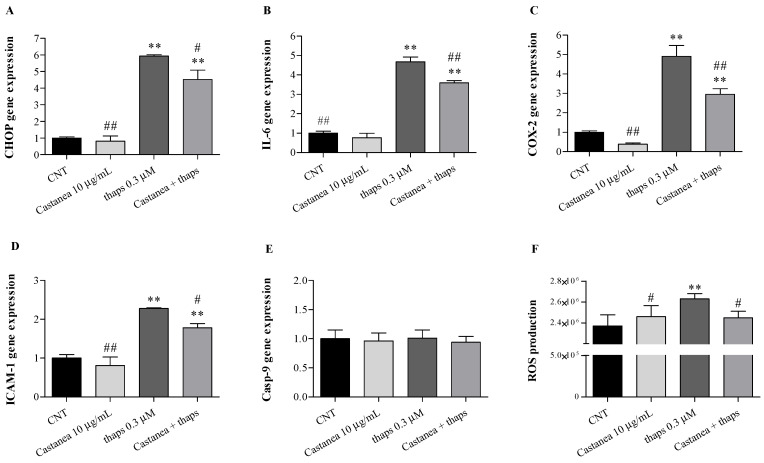
Effects of 1 h pre-treatment with 10 µg/mL *Castanea* bee pollen on HMEC-1 exposed 2 h to 0.3 µM thapsigargin (thaps): (**A**) CHOP, (**B**) IL-6, (**C**) COX-2, (**D**) ICAM-1, and (**E**) Casp-9 (Real-Time qPCR); (**F**) cellular ROS production (DCFH-DA assay). One-way ANOVA with Dunnett’s multiple comparison test: * significantly different from control (CNT): ** *p* < 0.01. # significantly different from thaps 0.3 μM: # *p* < 0.05; ## *p* < 0.01.

Our results demonstrated that HMEC-1 cell viability was not affected by *Castanea* bee pollen, whereas low cytotoxicity was observed at higher thapsigargin concentrations (1 and 3 μM, data not shown). Therefore, following 1 h pretreatment with increasing concentrations of *Castanea* bee pollen, HMEC-1 cells were stimulated for 2 h with or without 0.3 μM thapsigargin. Overall, our results demonstrated that thapsigargin treatment induced ER stress (Figure 3A) and up-regulated IL-6, COX-2, and ICAM-1 gene expression (Figure 3B–D). In addition, we found no alterations in Casp-9 expression following all treatments (Figure 3E). Moreover, a significant ROS overproduction was observed following thapsigargin exposure (Figure 3F).

It is known that the thapsigargin elicits UPR response and activates the NF-kB (nuclear factor kappa-light-chain-enhancer of activated B cells) pathway through ER calcium efflux, followed by the formation of the mitochondrial reactive oxygen intermediates (ROI). However, pre-incubation with calcium chelators and antioxidants can block the NF-kB activation [11]. Among others, free radicals have been recognized as crucial linkers responsible for integrating metabolic, inflammatory, and ER-stress responses [13].

In keeping with such evidence, *Castanea* bee pollen was effective in counteracting thapsigargin-induced microvascular endothelial cell alterations by reducing CHOP, IL-6, COX-2, and ICAM-1 genes over-expression. In addition, *Castanea* bee pollen pretreatment was effective to reduce the oxidation process arising from the thapsigargin exposure, with a maximum protective effect at 10 µg/mL (Figure 3A–F), while showing pro-oxidant effects at higher doses (100 and 200 µg/mL) (data not shown). Indeed, as demonstrated by Moita et al. [32], bee pollen extract from *Echium plantagineum* L. was able to scavenge the reactive oxygen species, both superoxide and nitric oxide radicals, and reduce markers of oxidative stress in a lipopolysaccharide-stimulated murine macrophage cell line (RAW 264.7) at low doses, being pro-oxidant at higher concentrations.

Oxidative stress, inflammation, and endothelial dysfunction are closely related to the etiology of numerous cardiometabolic diseases. Moreover, an imbalance between the generation of ROS and the antioxidant defense system represents the main cause of endothelial dysfunction, which leads to important vascular damage in both metabolic and atherosclerotic diseases [33]. However, a healthy eating approach can help reduce this imbalance.

To date, many phytochemicals and bioactive food components have been shown to be independently or jointly responsible for the apparent reduction in cardiovascular diseases risk, also by affecting and ameliorating endothelial functions [34]. Therefore, the use of bioactive phytochemicals in nutrition with a proven effect against oxidative stress, inflammation, and endothelial dysfunction represents a winning strategy to reduce the onset and progression of numerous cardiovascular and metabolic diseases.

Based on our preliminary results, bee pollen may represent a potential nutraceutical product useful both in clinical research and medical practice. However, further investigations on bee pollen bioactive component bio-accessibility, absorption in the gastrointestinal tract, and in vivo bioavailability are needed.

## 4. Conclusions

Our results showed good bee pollen antimicrobial potential with different selectivity among the Gram-negative and Gram-positive tested bacteria. In addition, bee pollen samples exhibit a significantly higher cellular antioxidant activity following all bee pollen pretreatments and better erythrocyte hemolysis protection by *Castanea* and *Cistus* samples, suggesting good ex vivo biological activity as free radical scavengers and natural antioxidants. Further, *Castanea* bee pollen was effective in counteracting ER stress and the oxidation process arising from the thapsigargin exposure by attenuating, at low concentration, all thapsigargin-induced HMEC-1 alterations. In conclusion, bee pollen represents a good natural antibacterial and antioxidant product, as well as an excellent food supplement and a valuable product with potential for medical and nutrition applications in the prevention of free radicals and ER stress-associated diseases. Further analyses, focusing on bee pollen samples’ comparison of different botanical origins and from different geographical areas, will be necessary to better understand how much our results are strictly dependent on the botanical origin or may be affected by other factors.

## Figures and Tables

**Table 1:** Minimum inhibitory concentration (MIC) values of *Castanea, Cistus*, and *Rubus* bee-pollen extracts on selected pathogen strains growth (O.D. 600 nm). Gentamicin and vancomicyn were used as target antibiotics.

		MIC Values		
Gram negative strains	*Gentamicin*	*Castanea*	*Cistus*	*Rubus*
*Escherichia coli*	0.05 mg/mL	10 mg/mL	10 mg/mL	-
*Salmonella typhimurium*	0.05 mg/mL	10 mg/mL	10 mg/mL	-
*Enterobacter erogene*	0.05 mg/mL	-	10 mg/mL	-
Gram positive strains	*Vancomycin*	*Castanea*	*Cistus*	*Rubus*
*Enterococcus faecalis*	0.05 mg/mL	-	5 mg/mL	10 mg/mL
*Staphylococcus aureus*	0.05 mg/mL	10 mg/mL	5 mg/mL	10 mg/mL

## Data Availability

The data presented in this study are available on request from the corresponding author.

## Supplementary Materials

The following are available online at https://www.mdpi.com/article/10.3390/foods10061422/s1, Table S1: Growth of selected pathogen strains in the presence of increasing concentrations (1, 2.5, 5, and 10 mg/mL) of *Castanea, Cistus,* and *Rubus* bee pollen extracts.
