# Supplementary material for: Antimicrobial Activity and Protective Effect of Tuscan Bee Pollens on Oxidative and Endoplasmic Reticulum Stress in Different Cell-Based Models
Source: Foods. 2021 Jun 18;10(6):1422. doi: 10.3390/foods10061422 (PMC8235197; doi:10.3390/foods10061422)
Supplement: Supplementary file 1 [file foods-10-01422-s001.zip › foods-1225095-supplementary.pdf]

Supplementary materials

Table S1. Growth of selected pathogen strains in the presence of increasing concentrations (1, 2.5, 5, and 10 mg/mL) of *Castanea, Cistus,* and *Rubus* bee pollen extracts.

	***Castanea* extract concentrations (mg/mL)**				
**Strains**	0	1	2.5	5	10
Escherichia coli ATCC 25922	1.20±0.02	0.80±0.02	0.72±0.02	0.60±0.02	**0.10±0.002**
Salmonella typhimurium ATCC 14028	1.22±0.03	0.90±0.01	0.68±0.02	0.70±0.03	**0.10±0.005**
Enterobacter aerogenes ATCC 13048	1.33±0.02	1.14±0.03	0.50±0.13	0.50±0.03	0.50±0.01
Enterococcus faecalis ATCC 29212	1.28±0.02	0.86±0.02	0.37±0.01	0.44±0.03	0.54±0.03
Staphylococcus aureus ATCC 25923	1.35±0.05	0.81±0.02	0.48±0.01	0.54±0.03	**0.10±0.003**

	***Cistus* extract concentrations (mg/mL)**				
**Strains**	0	1	2.5	5	10
Escherichia coli ATCC 25922	1.30±0.02	0.88±0.02	0.60±0.02	0.50±0.02	**0.07±0.002**
Salmonella typhimurium ATCC 14028	1.20±0.03	1.10±0.02	0.80±0.02	0.60±0.03	**0.08±0.003**
Enterobacter aerogenes ATCC 13048	1.35±0.02	1.20±0.03	0.90±0.10	0.50±0.03	**0.09±0.001**
Enterococcus faecalis ATCC 29212	1.27±0.03	0.75±0.02	0.57±0.01	**0.08±0.002**	0.05±0.003
Staphylococcus aureus ATCC 25923	1.40±0.05	0.90±0.02	0.50±0.01	**0.06±0.003**	0.03±0.002

	***Rubus* extract concentrations (mg/mL)**				
**Strains**	0	1	2.5	5	10
Escherichia coli ATCC 25922	1.20±0.02	0.83±0.02	0.60±0.02	0.50±0.03	0.45±0.002
Salmonella typhimurium ATCC 14028	1.10±0.03	0.90±0.02	0.70±0.02	0.55±0.02	0.60±0.003
Enterobacter aerogenes ATCC 13048	1.30±0.02	1.10±0.03	0.80±0.05	0.50±0.03	0.45±0.01
Enterococcus faecalis ATCC 29212	1.28±0.03	0.80±0.02	0.60±0.02	0.35±0.03	**0.10±0.002**
Staphylococcus aureus ATCC 25923	1.35±0.05	0.95±0.02	0.60±0.01	0.30±0.03	**0.07±0.001**

Bacterial growth (O.D. 600 nm). Results are expressed as mean ± SD.
